# Risk factors for postoperative ileus after diverting loop ileostomy closure

**DOI:** 10.1186/s12893-022-01583-2

**Published:** 2022-04-07

**Authors:** Toshihiro Nakao, Mitsuo Shimada, Kozo Yoshikawa, Takuya Tokunaga, Masaaki Nishi, Hideya Kashihara, Chie Takasu, Yuma Wada, Toshiaki Yoshimoto, Syoko Yamashita, Yosuke Iwakawa

**Affiliations:** Department of Digestive and Transplant Surgery, Tokushima University Hospital, 3-18-15 Kuramoto-cho, Tokushima City, Tokushima 7708503 Japan

**Keywords:** Postoperative ileus, Ileostomy closure, Diverting loop ileostomy, Risk factor, Rectal cancer

## Abstract

**Background:**

Postoperative ileus is one of the most common complications after diverting loop ileostomy closure. Some reports have investigated the risk factors for postoperative complications or ileus after ileostomy closure; however, these studies did not evaluate the index surgery sufficiently. In this study, we evaluated the risk factors, including the details of the index surgery, for ileus after diverting ileostomy closure.

**Methods:**

This was a retrospective study of patients who underwent ileostomy closure following index surgery for rectal cancer. Patients who developed postoperative ileus [POI (+)] and patients who did not [POI (−)] after ileostomy closure were compared.

**Results:**

Sixty-eight patients were evaluated and were divided into two groups: POI (+) (n = 11) and POI (−) (n = 57), and the groups were compared. There were no significant differences in the details of the index surgery, operative procedure, transanal total mesorectal excision, lateral lymph node dissection, operating time, or blood loss. The incidence of Clavien–Dindo grade ≥ III complications and adjuvant chemotherapy after index surgery were significantly higher in the POI (+) group.

**Conclusions:**

The incidence of Clavien–Dindo grade ≥ III complications and adjuvant chemotherapy after index surgery may increase the risk of postoperative ileus after ileostomy closure.

## Background

Anastomotic leakage is one of the most serious complications after rectal cancer surgery. Various methods have been used to prevent anastomotic leakage, such as combined mechanical and oral antibiotic bowel preparation, transanal tube placement, and evaluating the anastomosis with the indocyanine green (ICG) fluorescence method [1–3]. A diverting loop ileostomy is often performed for cases with a higher risk of developing anastomotic leakage [4]. Newer surgical techniques and devices have been developed, such as transanal total mesorectal excision (TaTME), that make it possible to anastomose for rectal cancer near the anus, and these techniques and developments have increased the indications for diverting loop ileostomy to prevent anastomotic leakage. Generally, the ileostomy is closed a few weeks or months after the surgery. Even though ileostomy closure is a relatively easy surgery, several postoperative complications often arise [5]. In particular, postoperative ileus is one of the most common complications.

We have performed diverting loop ileostomy in rectal cancer surgery for patients with a higher risk of developing anastomotic leakage. The risk factors for anastomotic leakage are male sex, high body mass index (BMI), high American Society of Anesthesiologists physical status (ASA-PS), large tumor size, preoperative chemotherapy, low-level anastomosis, multiple stapler firings, long operative time, high intra-operative transfusion/blood loss, and lack of a pelvic drain. We usually close the ileostomy from 3 to 6 months after the surgery [6].

Some reports have investigated the risk factors for postoperative complications or ileus after ileostomy closure [7–10]. However, these studies did not focus on the details of the index surgery, such as TaTME and lateral lymph node dissection (LLND). In this study, we evaluated the risk factors, including the details of the index surgery, for ileus after diverting ileostomy closure.

## Methods

### Patients

This was a retrospective study of patients who underwent diverting ileostomy closure in Tokushima University Hospital between January 2017 and December 2021. The endpoint of this study was to find the risk factors of postoperative ileus following after diverting ileostomy closure. The protocol was approved by the Ethics Committee of Tokushima University (approval number. 3215-1), and the study was conducted following the provisions of the Declaration of Helsinki. All patients provided informed consent for the use of their data. Diverting loop ileostomy was performed when the patients underwent surgery for rectal cancer, gastrointestinal stromal tumor, or neuroendocrine tumor. Anterior resection (AR), intersphincteric resection (ISR), total colectomy (TC), or transanal minimally invasive surgery (TAMIS) was performed for rectal tumors, and all surgeries were performed laparoscopically. Performing TaTME and LLND depended on the patient and tumor progression. Some of the patients diagnosed with stage II–IV lower rectal cancer underwent adjuvant radiotherapy, and some diagnosed with stage III or IV rectal cancer underwent adjuvant chemotherapy or neoadjuvant chemotherapy, respectively, depending on the patient’s condition. Exclusion criteria were (1) the patients who underwent diverting colostomy; (2) the patients who underwent diverting ileostomy for disease except for malignant gastrointestinal tumors, such as a gynecologic tumor, rectal injury, or anal fistula. Patients were divided into two groups: a POI (+) group, which represented patients who developed postoperative ileus after ileostomy, and a POI (−) group, which represented patients who did not develop postoperative ileus.

### Ileostomy closure

The ileostomy was closed between 1 and 25 months postoperatively. Ileostomies in patients receiving adjuvant chemotherapy were closed after completing adjuvant chemotherapy. All patient was confirmed that there was no recurrence by computed tomography (CT) and tumor marker. All patients underwent water-soluble contrast enema, colonoscopy from anus and stoma to check the patency and integrity of the anastomosis and distal limb of ileostomy before ileostomy closure. Food intake was stopped, and mechanical bowel preparation was performed the day before surgery. Our standard operative technique for ileostomy closure began with a peristomal skin incision, then the proximal and distal limbs were dissected to the peritoneal cavity, and the loop was resected, followed by anastomosis.

The reconstruction method was end-to-end anastomosis or side-to-side anastomosis by hand-sewn. The peritoneum and fascia were closed with interrupted sutures, and the skin was closed with a purse-string closure or linear closure. The selection of the anastomosis methods and skin closure methods depended on the surgeon’s preference. Antimicrobial prophylaxis was used from just before surgery until the first day after surgery, drinking water was started from the first day after surgery, and food intake was started from the third day after surgery. Patients were discharged on the eighth day or later after surgery, if there were no problems.

### Definition of postoperative ileus

Postoperative ileus was defined as a temporary impairment in gastrointestinal motility following surgery. The criteria of postoperative ileus were digestive symptoms, such as abdominal pain and distension, with nausea and vomiting, and X-ray and CT findings, such as intestinal dilation and multiple air-fluid levels throughout the abdomen on or after postoperative day 3. Small bowel obstruction was excluded by checking that there was no caliber change or closed loop.

### Statistical analysis

The collected patient data were reviewed. All statistical analyses were performed with EZR (Version 1.54) (Saitama Medical Center, Jichi Medical University, Saitama, Japan), a graphical user interface for R (version 4.03) (R Foundation for Statistical Computing, Vienna, Austria) [11]. More precisely, EZR is a modified version of R commander (version 2.7-1) designed to add statistical functions used frequently in biostatistics. Categorical variables were analyzed with Fisher’s exact test, and continuous variables were analyzed with Student’s t-test. A *p* value of less than 0.05 was considered statistically significant.

## Results

The patients’ characteristics are summarized in Table 1. A total of 68 patients, 48 (70.6%) male and 20 (29.4%) female, underwent closed ileostomy between January 2017 and December 2021. The compliance of the patients during the study was good, and there was no loss of follow-up patients. The mean age was 64.4 years, and the mean body mass index was 22.7 kg/m^2^. Twenty-four (35.3%), 37 (54.4%), and 7 (10.3%) patients had ASA-PS I, II, and III, respectively. The primary diseases were rectal cancer [stage 0: 1 (1.5%), I: 27 (39.7%), II: 17 (25.0%), III: 15 (22.0%), IV: 5 (7.3%), and unknown: 1 (1.5%)], gastrointestinal stromal tumor [1 (1.5%)], and neuroendocrine tumor [1 (1.5%)]. The tumors were located rectosigmoid (Rs) [17 (25.0%)], rectum above the peritoneal reflection (Ra) [20 (29.4%)] and rectum below the peritoneal reflection (Rb) [31 (45.6%)]. Laparoscopic AR, ISR, TC, and TAMIS were performed for 45 (66.2%), 20 (29.4%), 2 (2.9%), and 1 (1.5%) patients, respectively. The anastomosis level of AR was as follows; 7 (15.6%) were above peritoneal reflection and 38 (84.4%) were below the peritoneal reflection. Among the procedures, TaTME was performed for 36 (52.9%) patients, and LLND was performed for 15 (22.1%) patients. The mean operating time for the index surgery was 352.6 min, and the mean blood loss was 61.4 ml. Complications (Clavien–Dindo grade ≥ III) developed in 5 (7.4%) patients after the index surgery. Complications comprised two grade IIIa ileus, two grade IIIa anastomotic stenosis, and 1 grade IIIb neurogenic bladder. Four (5.9%) patients underwent neoadjuvant radiotherapy, and 15 (22.1%) patients underwent neoadjuvant chemotherapy; 20 (29.4%) patients underwent adjuvant chemotherapy. The interval between the index surgery and ileostomy closure was 181.5 days. The ileostomy was resected and anastomosed. End-to-end anastomosis was performed for 62 (91.2%) patients, and side-to-side anastomosis was performed for 6 (8.8%) patients. The skin was closed with a purse-string closure in 64 (94.1%) patients and with a linear closure in 4 (5.9%) patients. The mean operating time was 94.0 min, and the mean blood loss was 12.3 ml. Eleven (16.2%) patients developed ileus after surgery. Among them, 2 (18.2%) patients were Clavien–Dindo grade I, 3 (27.3%) patients were grade II, 4 (45.4%) patients were grade IIIa, and 1 (9.1%) patient was grade IIIb. The mean duration of treatment for postoperative ileus was 7.9 days. No patients developed anastomotic leakage or wound infection. Food intake has started at a mean of 6.5 days after surgery, and the mean hospital stay after surgery was 11.9 days.

**Table 1:** Patients’ characteristics and comparison between the POI (+) group and POI (−) group

	Total (n = 68)	POI (+) (n = 11)	POI (−) (n = 57)	p value
Age (years)	64.4 ± 10.0	69.5 ± 7.7	63.4 ± 10.3	0.065
Sex (Male/Female)	48/20	9/2	39/18	0.487
ASA-PS (I/II/III)	24/37/7	3/6/2	21/31/5	0.636
BMI	22.7 ± 3.4	23.0 ± 2.3	22.6 ± 3.6	0.735
Cancer				
Rotation (Rs/Ra/Rb)	17/20/31	14/24/19	3/1/7	0.235
Stage (0/I/II/III/IV/X/others)	1/27/17/15/5/1/2	1/6/2/0/1/0/1	0/21/15/15/4/1/1	0.068
Index surgery				
Laparoscopic operative procedure (AR/ISR/TC/TAMIS)	45/20/2/1	6/3/1/1	39/17/1/0	0.113
TaTME	36 (52.9%)	6 (54%)	30 (52.6%)	1.000
LLND	15 (22.1%)	2 (18.2%)	13 (22.8%)	1.000
Operating time (min)	352.6 ± 116.4	383.7 ± 144.4	346.6 ± 111,8	0.341
Blood loss (ml)	61.4 ± 75.9	69.9 ± 63.2	60.0 ± 79.2	0.726
Complication (Clavien–Dindo grade ≥ III)	5 (7.4%)	3 (27.3%)	2 (3.5%)	0.022
Ileus	2 (2.9%)	1 (9.1%)	1 (1.8%)	0.299
Anastomotic stenosis	2 (2.9%)	1 (9.1%)	1 (1.8%)	0.299
Neurogenic bladder	1 (1.5%)	1 (9.1%)	0 (0%)	0.169
Neoadjuvant radiotherapy	4 (5.9%)	1 (9.1%)	3 (5.3%)	0.515
Neoadjuvant chemotherapy (Yes/No)	15 (22.1%)	1 (9.1%)	14 (24.6%)	0.433
Adjuvant chemotherapy (Yes/No)	20 (29.4%)	0 (0%)	20 (35.1%)	0.026
Interval between the index surgery and ileostomy closure (days)	183.5 ± 125.3	143.9 ± 28.8	192.8 ± 135.5	0.165
Ileostomy closure				
Anastomosis (end-to-end/side-to-side)	62/6	11/0	51/6	0.579
Skin closure (purse string/linear)	64/4	11/0	53/4	1.000
Operating time (min)	94.0 ± 24.3	98.7 ± 14.1	93.1 ± 26.0	0.488
Blood loss (ml)	12.3 ± 22.1	6.4 ± 8.7	13.5 ± 23.8	0.334
Complication	16 (22.1%)			
Ileus	11 (16.2%)	11 (100%)	NA	NA
Clavien–Dindo grade I	2 (18.2%)	2 (18.2%)	NA	NA
Clavien–Dindo grade II	3 (27.3%)	3 (27.3%)	NA	NA
Clavien–Dindo grade IIIa	5 (45.4%)	5 (45.4%)	NA	NA
Clavien–Dindo grade IIIb	1 (9.1%)	1 (9.1%)	NA	NA
Duration of treatment (days)	7.9 ± 4.5	7.9 ± 4.5	NA	NA
Other	5 (7.4%)	1 (9.1%)	4 (7.0%)	0.813
Food intake after surgery (days)	6.5 ± 5.2	12.4 ± 5.0	5.4 ± 4.5	> 0.001
Hosptal stay after surgery (days)	11.9 ± 4.2	18.5 ± 5.6	10.6 ± 2.5	> 0.001

*ASA-PS* American Society of Anesthesiologists physical status, *BMI* body mass index, *Rs* Rectosigmoid, *Ra* rectum above the peritoneal reflection, *Rb* rectum below the peritoneal reflection, *AR* anterior resection, *ISR* intersphincteric resection, *TC* total colectomy, *TAMIS* transanal minimally invasive surgery, *TaTME* transanal total mesorectal excision, *LLND* lateral lymph node dissection

The POI (+) group and the POI (−) group were compared (Table 1). There were 11 patients in the POI (+) group and 57 patients in the POI (−) group. There was no significant difference in age, sex, ASA-PS, BMI, tumor location, or cancer stage between the groups. There was no significant difference in operative procedure for the index surgery (*p* = 0.113). There was also no difference in operating time and blood loss for the index surgery between the groups. In the POI (+) group, the incidence of Clavien–Dindo grade ≥ III complications after the index surgery was significantly higher than in the POI (−) group (*p* = 0.022). There was no difference regarding neoadjuvant radiotherapy or neoadjuvant chemotherapy for the primary disease. However, adjuvant chemotherapy was administered significantly more often in the POI (−) group compared with the POI (+) group (*p* = 0.026). The interval between the index surgery and ileostomy closure did not differ between the groups. Regarding the ileostomy closure technique, there was no significant difference between end-to-end anastomosis and side-to-side anastomosis, and there was no significant difference between purse-string skin closure and linear skin closure between the groups. Operating time and blood loss did not differ significantly between the groups regarding the surgical outcomes. Food intake was later, and hospital stay was longer, in the POI (+) group than in the POI (−) group.

## Discussion

Ileus is one of the most common postoperative complications after ileostomy closure, with a reported incidence of 16.4–33.0%. Postoperative ileus developed in 4.9–16.8% of patients after ileostomy closure [7, 9, 10]. In this study, the incidence of postoperative complications after ileostomy closure was 22.6%, and the incidence of ileus was 14.5%.

Some reports have investigated the risk factors for postoperative complications or ileus after ileostomy closure [7–10]. However, these studies did not focus on the details of the index surgery, and the relationship between the index surgery and postoperative ileus after ileostomy closure has not been sufficiently evaluated. Laparotomy and total colectomy during the index surgery are reported risk factors for postoperative ileus after ileostomy [8]. In the present study, all index surgeries were performed laparoscopically, and there was a significant difference in laparoscopic operative procedures between the POI (+) and POI (−) groups. There was no significant difference in TaTME, LLND, operating time, and blood loss between the groups. In this study, Clavien–Dindo grade ≥ III complications of index surgery were significantly more common in the POI (+) group, and it was an independent predictive factor for postoperative ileus following after diverting ileostomy closure. Additionally, grade IIIa ileus, grade IIIa anastomotic stenosis, and grade IIIb neurogenic bladder occurred in the POI (+) group; the patient who developed neurogenic bladder underwent cystostomy. Previous studies reported the risk factors for postoperative ileus as low albumin, opioid use, long duration of surgery, emergency surgery, and blood loss requiring transfusion [12]. Severe complications sometimes cause some of these factors, and prolonged bed rest or invasive treatment for the severe complications might induce intestinal adhesion. These factors might be related to the incidence of postoperative ileus after ileostomy closure.

Previous studies also reported that neoadjuvant radiotherapy, neoadjuvant chemotherapy, and adjuvant chemotherapy did not affect the incidence of postoperative ileus after ileostomy closure [9]. In the present study, there was no difference in neoadjuvant radiotherapy and chemotherapy administration between the POI (+) and POI (−) groups. However, significantly more patients received adjuvant chemotherapy in the POI (−) group than in the POI (+) group. The indication for adjuvant chemotherapy was stage III or IV rectal cancer, and there was no difference in the rate of adjuvant chemotherapy between patients with stage III vs IV rectal cancer. Cancer progression because of not undergoing adjuvant chemotherapy might contribute to the development of postoperative ileus.

This study suggested that there was no relationship between the interval between the index surgery and ileostomy closure and postoperative ileus after ileostomy closure [13]. A previous study also reported that postoperative complication was similar in the early ileostomy closure group and late closure group [14].

Regarding the ileostomy closure technique, there was no significant difference in anastomotic leakage rates between hand-sewn and stapled techniques in one study, but the rate of small bowel obstruction and ileus were significantly lower with the stapled technique [15]. In the present study, 91.2% of the patients underwent end-to-end anastomosis, and 8.8% of patients underwent side-to-side anastomosis; all but one of the anastomoses were hand-sewn. There was no significant difference in the rate of ileus between end-to-end and side-to-side anastomosis, and between the hand-sewn and stapled techniques.

Food intake was later, and hospital stay was longer, in the POI (+) group than in the POI (−) group in this study. It considered that the treatment of ileus delayed the start of meals and extended the length of hospital stay.

There were some limitations in this study. First, the study is a retrospective study. There are selection and information biases. All data that might affect the development of postoperative ileus could not be obtained. Second, the small sample size is due to a single-center study. The small sample size may inhibit our ability to detect differences where differences exist, such as the effect of other factors on ileus. However, additional cases would only likely serve to strengthen the effect of the index operation complications on ileus. The small sample size also prevented multivariate analysis. Multivariate analysis is needed to identify true independent risk factors. Third, there was a bias of index surgery and adjuvant chemotherapy. No patient underwent adjuvant chemotherapy in POI (+) group. It may affect the accuracy of the analysis. A multi-center prospective study is needed to overcome these limitations.

## Conclusions

Postoperative ileus is a common complication after diverting loop ileostomy closure. Complications and adjuvant chemotherapy after index surgery may increase the risk of postoperative ileus after ileostomy closure.

## Data Availability

The datasets used and/or analyzed during the current study are available from the corresponding author on reasonable request.

## Abbreviations

AR: Anterior resection
ASA-PS: American Society of Anesthesiologists physical status
BMI: Body mass index
CT: Computed tomography
ICG: Indocyanine green
ISR: Intersphincteric resection
LLND: Lateral lymph node dissection
TAMIS: Transanal minimally invasive surgery
TaTME: Transanal total mesorectal excision
TC: Total colectomy
